# Relationship between tea intake and cedar pollen allergy: a population-based cross-sectional study

**DOI:** 10.1017/jns.2024.96

**Published:** 2025-01-10

**Authors:** Nozomi Aoki, Mai Suzuki, Yuki Sato, Hirohide Yokokawa, Toshio Naito

**Affiliations:** 1 Department of General Medicine, Juntendo University Faculty of Medicine, Tokyo, Japan; 2 Department of Occupational Epidemiology, National Institute of Occupational Safety and Health, Kiyose, Japan

**Keywords:** cedar pollen, Green tea, IgE, pollinosis, tea drinking, BMI, body mass index, CIs, 95% confidence intervals, ELISA, enzyme-linked immunosorbent assay, IgE, immunoglobulin E, LC, lumicount, LDL, low density lipoprotein, OR, sodds ratios, ToMMo, Tohoku Medical Megabank Organization

## Abstract

Tea intake has been associated with health benefits, including potential beneficial effects of catechin-containing teas on allergic symptoms. However, large-scale epidemiological studies on the relationship between tea intake and allergic symptoms have been limited. The present study aimed to examine the relationship between the frequency of tea intake and cedar pollen allergy, which is a major cause of seasonal hay fever in Japan, in a large Japanese epidemiological cohort. Data on cedar pollen antibody levels assessed by blood tests and frequency of tea intake (green tea, coarse tea, oolong tea, and black tea) by a self-administered questionnaire from 16,623 residents in the Tohoku region of Japan were used in this study. The association between frequency of tea intake (less than once a week, 1–6 times/week, and more than once a day) and serum levels of cedar pollen-specific IgE (lumicount, LC: negative, 0–1.39; positive, ≥1.40) was analysed using a logistic regression model. Green tea intake (≥vs. <1/day) was inversely associated with cedar pollen-specific IgE (adjusted OR = 0.81, 95% CI, 0.70, 0.94). No statistically significant association between cedar pollen-specific IgE and frequency of tea intake was found for other types of tea. Our results suggest that green tea intake may be associated with lower cedar pollen-specific IgE positivity.

## Introduction

The number of patients with allergic rhinitis, conjunctivitis, and other symptoms caused by pollen has been increasing in Japan, and hay fever is one of the most common health concerns worldwide^(1)^. Symptoms such as allergic rhinitis decrease work efficiency, cause sleep disturbance and depression, and decrease academic performance due to poor concentration in children^(2–4)^. Seasonal allergies are often caused by ragweed and grass in Europe and the United States, while cedar is the major cause in Japan. In Japan, cedar (*Cryptomeria japonica D*.) trees were planted nationwide from around the 1970s for forestry purposes, and the amount of cedar pollen dispersal has increased year by year as these trees have grown. Hence, the planting of cedar trees in the past is considered the main reason for the recent increase in the number of pollinosis patients in Japan^(5)^. The prevalence of hay fever in Japan was 42.5% in 2019, having increased by 10% every preceding decade. In particular, the prevalence of cedar pollen allergy was 38.8% in 2019. That is, one in three Japanese people suffer from cedar pollinosis, public concern to prevent cedar pollen allergy is high and an important matter. The north-eastern region of Japan, where data for the present study were collected, is a region with high levels of cedar pollen. The number of hay fever sufferers in this region being slightly lower than in large metropolitan areas in Japan, and the number of sufferers has been increasing every year^(6)^.

Green tea is currently consumed worldwide, and drinking green tea has been an important part of Japanese culture^(7)^. Many studies have been conducted on the health-promoting and disease-preventing effects of green tea^(8–18)^. For instance, green tea has been shown to have an effect on low-density lipoprotein (LDL) cholesterol levels^(10)^, prevention of type 2 diabetes^(11)^, obesity^(12)^, and other lifestyle-related diseases and cardiovascular diseases^(13)^, dementia and brain function^(14)^, prevention of infectious disease^(15)^ and anti-cancer effects^(16)^. Two epidemiological studies have reported that green tea intake may reduce mortality due to its preventive effects on various diseases, such as heart and cerebrovascular disease, cancer, and respiratory disease^(17,18)^. One study reported that Japanese cultivars of green tea are effective against seasonal allergic rhinitis^(19)^. The mechanism underlying the beneficial effects of green tea on health appears to derive from anti-inflammatory effects due to antioxidant actions of substances found in tea, such as catechins^(20)^. These components inhibited histamine release, thereby alleviating hay fever symptoms and atopic dermatitis in mice^(21,22)^. The effects of other types of tea on allergies have also been noted, reports from large-scale epidemiological studies have been also limited.

Hence, the present study examined the relationship between the intake of four types of tea and cedar pollen allergy, a major seasonal allergy in Japan, in a large Japanese epidemiological cohort.

## Methods

### Study population

**Figure 1.**
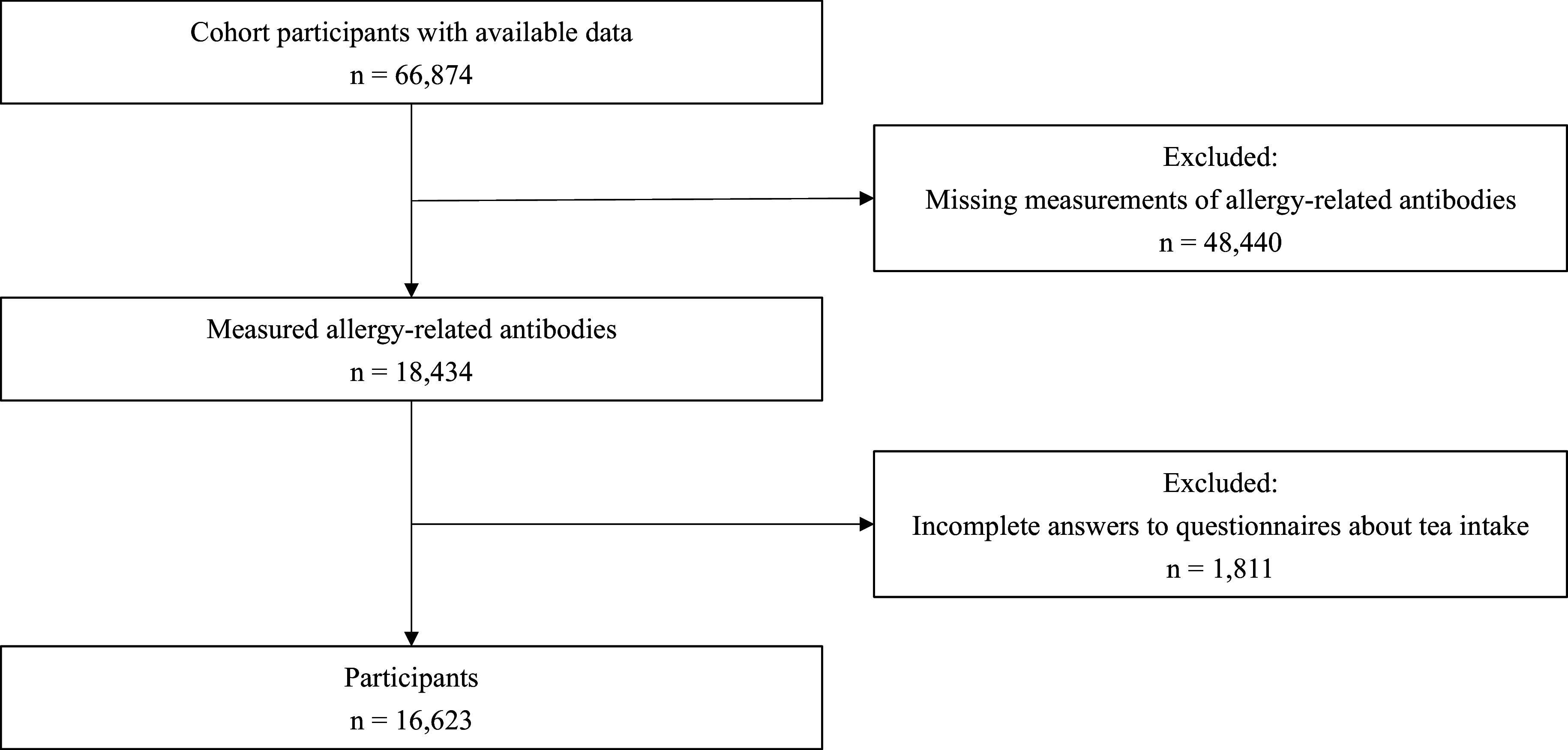
Flow chart for participants selection.

Participants of the present study were enrolled in the Tohoku Medical Megabank Community-Based Cohort Study. Details of the cohort study have been described elsewhere^(23,24)^. Briefly, the cohort includes approximately 60,000 participants aged about ≥40 years, who live in Miyagi and Iwate prefectures in the north-eastern part of Japan, and who were recruited and participated in a health survey between 2013 and 2015. The health survey included self-administered questionnaires, physical measurements, and biochemical tests of blood and urine. The present study used cross-sectional data of cohort participants who had available measurements for the cedar pollen antibody blood test and who had completed the questionnaire about tea intake(*n* = 16,623). A flow chart summarising participant selection is shown in Fig. 1.

### Data variables

#### Tea intake

Habitual tea intake was assessed using a self-administered questionnaire, which asked about the intake of four types of tea: green tea, coarse tea (*bancha*), oolong tea, and black tea. Response options for frequency were as follows: never or less than once a week, 1–2 times a week, 3–4 times a week, 5–6 times a week, 1 cup daily, 2–3 cups daily, 4–6 cups daily, 7–9 cups daily, and ≥10 cups daily.

#### Cedar pollen allergy

Cedar pollen allergy status was determined by cedar pollen antibody levels. Allergen-specific serum immunoglobulin E (IgE) antibodies (hereafter, cedar pollen-specific IgE) were measured by enzyme-linked immunosorbent assay (ELISA). Measured lumicount (LC) values were used as the level of allergic reactivity. The lower limit of measurement was 0 (zero), with higher values indicating higher antibody levels. Values between 0 and 1.39 were defined as a negative antibody response^(25,26)^. According to this criterion, participants with LC values of 0–1.39 were classified as ‘negative cases’ and those with LC values ≥1.40 as ‘positive cases’. The timing of blood sampling was not restricted to periods of particularly high cedar pollen dispersion. Measured values thus reflect the state of allergic reactions throughout the year. Therefore, distinctions were adopted in the present study such that even the slightest allergic reaction was judged to be positive.

#### Other variables

The following variables were considered confounding factors based on previous existing reports^(27–35)^. Data for sex, age, and anthropometric measurements (height, weight, and body mass index (BMI)) were collected at health surveys. BMI was calculated by dividing body weight (kg) by height squared (m^2^). Health status and lifestyle habits (alcohol drinking, smoking, physical activity, sleeping, and medication use for hypertension, diabetes mellitus, and dyslipidemia) were based on answers to the self-administered questionnaire.

## Statistical analysis

Results are presented as mean ± standard deviation for continuous variables or prevalence (%) for categorical variables. The *t*-test for continuous variables and the chi-square test or Fisher’s exact test for categorical variables were used to compare mean values and proportions between groups. Logistic regression analysis was used to calculate odds ratios (ORs) and 95% confidence intervals (CIs) for associations between tea intake frequency and cedar pollen antibody levels. The serum level of cedar pollen-specific IgE was used to define ‘positive cases’ (LC values of 0–1.39) and ‘negative cases’ (LC values ≥1.40). The frequency of tea intake was reclassified into three groups (less than once a week, 1–6 times a week, more than once a day). Analyses were conducted with no adjustment for confounding factors and in two adjusted models. Model 1 was adjusted for age (<65 years, ≥65 years) and sex (male, female). Model 2 was adjusted for age (<65 years, ≥65 years), sex (male, female), BMI (<25, ≥25), alcohol intake (daily, not daily), smoking (lifetime cumulative smoking of ≥100 cigarettes, <100 cigarettes), exercise frequency (walking at least once a week or less than once a week), sleep duration (6–7 hours/day, <6 hours and ≥8 hours/day), and medication use for hypertension, dyslipidemia, and/or diabetes (user, non-user). The interaction of the variables was examined using the variable reduction stepwise and forced entry method. All statistical analyses were performed using the Statistical Package for Social Sciences, IBM SPSS Statistics 27.0.

The present study was conducted in compliance with the Ethical Guidelines for Epidemiological Studies established by the Japanese government^(36)^ and the Declaration of Helsinki of 1975 (revised in 2000)^(37)^. The research protocol was reviewed and approved by the Ethics Committee of Juntendo University (E22-0457). The committee of ToMMo data use also reviewed and approved the research protocol (No. 2019-0079). Written informed consent was obtained from all participants. Consent to participate was additionally obtained by opt-out.

## Results

Table 1 summarises participant characteristics and compares these characteristics between negative and positive cases of cedar pollen-specific IgE. Overall, the mean age of participants was 60.3 years, 44.5% were aged ≥65 years, and 37.9% were male, with slightly more female participants. There was no extreme disproportion in characteristics between groups, with about half of participants consuming green tea more than once a day. The frequency of tea intake more than once a day was 51.6% for green tea, 23.1% for coarse tea, 6.5% for oolong tea, and 4.8% for black tea. Compared to positive cases of cedar pollen-specific IgE, negative cases tended to be older, more likely to be older than ≥65 years, slightly less likely to consume alcohol daily, slightly less likely to smoke, and slightly more likely to have a habit of walking at least once a week and to consume green tea or coarse tea at least once a day. Compared to the overall in Table 1 in this study, 5,983 people without data of tea intake and excluded from this study were higher percentages of age≥ 65 years (50.7%), male (49.3%), BMI ≥ 25 kg/m^2^ (38.3%), similar percentage of current smoke (38.3%), and lower percentages of daily alcohol intake (9.3%), physical activity of walking ≥1 time/week (8.0%), and sleep duration of 6–7 hours/day (12.4%), medication use for hypertension (11.3%), diabetes mellitus (2.9%), and dyslipidemia (12.5%). The 48,440 people did not have blood test data for cedar pollen IgE antibody titre and also excluded from this study were higher percentages of age≥ 65 years (47.4%), and male (38.7%), and lower percentage of BMI ≥ 25 kg/m^2^ (29.9%), daily alcohol intake (17%), current smoke (34.9%), physical activity of walking ≥1 time/week (22.2%), sleep duration of 6–7 hours/day (36.4%), medication use for hypertension (25.4%), diabetes mellitus (6.3%), and dyslipidemia (11.4%)., The results of logistic regression analysis are shown in Table 2. For green tea, the univariate OR (95% CI) for cedar pollen-specific IgE positivity was 0.70 (0.69–0.75) for an intake frequency of more than once a day (Group 3) when less than once a week (Group 1) was used as the reference; adjusted ORs were 0.82 (0.76–0.90) in Model 1 and 0.81 (0.70–0.94) in Model 2. For coarse tea, the univariate OR (95% CI) was 0.79 (0.73–0.85) for an intake frequency of more than once a day (Group 3) when less than once a week (Group 1) was used as the reference; adjusted ORs were 0.90 (0.83–0.97) in Model 1 and 1.02 (0.87–1.19) in Model 2. The results were similar for oolong tea, with a univariate OR (95% CI) of 0.83 (0.73–0.94) for an intake frequency of more than once a day (Group 3) when less than once a week (Group 1) was used as the reference; adjusted ORs were 0.85 (0.73–0.97) in Model 1 and 1.00 (0.78–1.26) in Model 2. For black tea, there was no association between the frequency of tea intake and cedar pollen-specific IgE positivity. When analysing linear trends, higher intake frequencies of green tea, coarse tea, and oolong tea were associated with lower odds of cedar pollen-specific IgE positivity (*p* for trend <0.01) (Data not shown). However, the number of categories may not have been sufficient for linearity testing because there were only three categories, including the reference category.

**Table 1.** Participant characteristics

	Cedar pollen allergy test^*^						
	All (*n* = 16,623)		Negative cases (*n* = 9,308)		Positive cases (*n* = 7,315)		*p* ^†^
Age, years, mean ± s.d.	60.3	± 0.9	62.3	± 9.8	57.6	± 12.1	<0.01
Age, ≥65 years	7,405	(44.5)	4,806	(51.6)	2,599	(35.5)	<0.01
Sex, male	6,306	(37.9)	3,499	(37.6)	2,807	(38.4)	<0.01
Body mass index, kg/m^2^, mean ± s.d.	23.6	± 3.5	23.7	± 3.5	23.4	± 3.6	0.03
Body mass index, ≥25 kg/m^2^	5,198	(31.3)	3,024	(32.5)	2,174	(29.4)	<0.01
Lifestyle characteristics							
Frequency of alcohol intake, daily	7,764	(46.1)	3,944	(42.4)	3,720	(50.9)	<0.01
Smoking, current smoker^‡^	6,360	(38.2)	3,418	(36.7)	2,942	(40.2)	<0.01
Physical activity, walking ≥1 time/week	5,176	(31.1)	2,484	(30.9)	2,692	(28.6)	<0.01
Sleep duration, 6–7 hours/day	8,929	(57.7)	6,212	(37.4)	2,717	(37.1)	0.05
Hypertension, medication user	6,263	(37.7)	4,518	(27.2)	1,745	(23.9)	<0.01
Diabetes mellitus, medication user	1,354	(8.1)	966	(5.8)	388	(5.3)	<0.01
Dyslipidemia, medication user	2,349	(14.1)	1,708	(10.3)	641	(8.8)	<0.01
Frequency of tea intake, more than once/day							
Green tea	8,579	(51.6)	5,110	(54.9)	3,469	(47.4)	<0.01
Coarse tea	3,841	(23.1)	2,305	(24.9)	1,536	(21.0)	<0.01
Oolong tea	1,087	(6.5)	631	(6.8)	456	(6.2)	<0.01
Black tea	800	(4.8)	450	(4.8)	350	(4.8)	0.25

Values in the table indicate the number of participants (percentage) or mean value ± standard deviation (s.d.).

* Participants with serum cedar pollen-specific IgE values (LC) of 0 to 1.39 were considered ‘negative cases’ and those with LC values above 1.40 were considered ‘positive cases’.

† Comparisons between negative and positive groups: *t*-test for continuous variables and chi-square test or Fisher’s exact test for categorical variables.

‡ Current smoker: currently smoking and lifetime cumulative smoking of ≥100 cigarettes.

**Table 2.** Odds ratios for positive serum cedar pollen-specific IgE by frequency of tea intake

	Number of negative cases^*^	Number of positive cases^*^	Univariate analysis		Multivariate analysis			
			OR (95% CI)	*p*	Model 1^†^ OR (95% CI)	*p*	Model 2^‡^ OR (95% CI)	*p*
*Types of tea and frequency of intake* ^§^								
**Green tea**	9,308	7,315						
Group 1	1,663	1,629	1.00 (reference)		1.00 (reference)		1.00 (reference)	
Group 2	2,535	2,217	0.89 (0.82–0.98)	0.13	0.93 (0.85–1.01)	0.11	0.94 (0.80–1.10)	0.40
Group 3	5,110	3,469	0.70 (0.69–0.75)	<0.01	0.82 (0.76–0.90)	<0.01	0.81 (0.70–0.94)	<0.01
**Coarse tea**	8,892	7,090						
Group 1	4,189	3,536	1.00 (reference)		1.00 (reference)		1.00 (reference)	
Group 2	2,398	2,018	1.00 (0.93–1.07)	0.34	1.01 (0.94–1.09)	0.74	1.08 (0.95–1.23)	0.26
Group 3	2,305	1,536	0.79 (0.73–0.85)	<0.01	0.90 (0.83–0.97)	<0.01	1.02 (0.87–1.19)	0.86
**Oolong tea**	7,331	6,202						
Group 1	4,496	3,915	1.00 (reference)		1.00 (reference)		1.00 (reference)	
Group 2	2,204	1,831	0.95 (0.89–1.03)	0.22	0.95 (0.88–1.03)	0.22	1.06 (0.93–1.21)	0.36
Group 3	631	456	0.83 (0.73–0.94)	<0.01	0.85 (0.73–0.97)	0.02	1.00 (0.78–1.26)	0.34
**Black tea**	6,886	5,962						
Group 1	4,702	4,075	1.00 (reference)		1.00 (reference)		1.00 (reference)	
Group 2	1,734	1,537	1.02 (0.94–1.11)	0.58	1.04 (0.96–1.13)	0.39	1.07 (0.92–1.23)	0.39
Group 3	450	350	0.90 (0.78–1.04)	0.15	0.95 (0.82–1.10)	0.51	1.27 (0.95–1.70)	0.10

OR, odds ratio, 95% CI; 95% confidence interval.

* Participants with serum cedar pollen-specific IgE values (LC) of 0 to 1.39 were considered ‘negative cases’ and those with LC values above 1.40 were considered ‘positive cases’.

† Model 1 was adjusted for age (<65 years, ≥65 years) and sex (male, female).

‡ Model 2 was adjusted for age (<65 years, ≥65 years), sex (male, female), BMI (<25, ≥25), alcohol intake (daily, not daily), smoking (lifetime cumulative smoking of ≥100 cigarettes, <100 cigarettes), physical activity (≥1 time/week for walking, <1 time/week for walking), sleep duration (6–7 hours/day, <6 hours and ≥8 hours/day), and medication use (hypertension, dyslipidemia, diabetes).

§ Three categories of tea intake frequency: Group 1, less than once a week; Group 2, 1–6 times a week; Group 3, more than once a day.

For green tea, further analysis was conducted in high intake categories (1 cup/day, 2–3 cups/day, 4–6 cups/day, 7–9 cups/day, and >10 cups/day). The results in the fully adjusted Model 2, ORs (95% CI, *p*-value) for cedar pollen-specific IgE positivity were 0.96 (0.79–1.17, *p* = 0.68) for 1 cup/day, 0.74 (0.62–0.89, *p* < 0.01) for 2–3 cups/day, 0.88 (0.71–1.10, *p* = 0.26) for 4–6 cups/day, 0.67 (0.44–1.00, *p* = 0.52) for 7–9 cups/day, and 0.52 (0.32–0.85, *p* < 0.01) for >10 cups/day (*p* for trend <0.01), as a reference to less than once a week (Data not shown).

In addition, the interaction analysis showed an effect of age group (*p* < 0.01) and no significant effect of other key variables such as sex (*p* = 0.25) and smoking habit (*p* = 0.49). Hence the sub-analysis by age group (≥65 years, <65 years) was conducted (Supplementary Table). The subgroup analyses showed statistically significant association between cedar pollen-specific IgE positivity and green tea intake frequency group G3 (more than once a day) in the <65 age group, the ORs (95%CI) were 0.75 (0.68–0.84, *p* < 0.01) in the univariate analysis, 0.76 (0.68–0.84, *p* < 0.01) in Model 1 and 0.81 (0.68–0.97, *p* = 0.02) in Model 2 of the multivariate analysis. In contrast, no statistically significant associations were shown for age ≥ 65 years.

## Discussion

The present epidemiological study found that habitual intake of tea more than once a day, particularly green tea, was significantly associated with a lower probability of cedar pollen-specific IgE positivity.

Several possible explanations exist for the association between green tea intake and allergic symptoms. One hypothesis suggests that the antioxidant effect of green tea acts on the immune system in allergic diseases^(21)^. Mechanisms leading to reduced IgE and histamine levels were linked to reduced expression of FcεRI, a regulating effect on the Th1/Th2/Th17/Treg cell balance, and the suppression of transcription factors related to allergic symptoms. The catechin content of tea differs by the type of tea. Catechins decrease as the fermentation manufacturing process of tea leaves takes longer, with the fermentation time shortest for green tea, followed by coarse tea, oolong tea, and black tea^(38)^. This difference in catechin content may explain the finding that green tea intake is inversely associated with cedar pollen-specific IgE positivity. Experimental studies on catechins have also reported that epigallocatechin-3-gallate in catechins inhibits IgE/Ag-induced activation of mouse mast cells; histamine release, leukotriene release, and cytokine production and secretion are all inhibited^(39)^, resulting in immunosuppressive alterations on human monocyte-derived dendritic cells, both by induction of apoptosis and suppression of cell surface molecules and antigen presentation^(40)^. Some reports have suggested the socio-environmental or age-related sensitivities to pollen allergy and immunity^(41,42)^. Cedar pollen-specific IgE titres increase as the amount of cedar pollen dispersed increases^(41)^. To interpret the association with the causes of pollinosis, environmental factors and immune status should also be considered. A patient-based clinical trial of hay fever reported that drinking green tea containing 34 mg/day of O-methylated catechin over 1.5 months before pollen dispersion significantly relieved nose-blowing and itchy eyes compared to drinking placebo green tea during the most severe cedar pollen dispersion period^(43)^. The present study did not examine annual variations in pollen distribution and individual pollen exposure. The amount of specific IgE in serum, which was examined in the present study, is one method for diagnosing the cause of allergic symptoms^(44)^, but it does not exactly correlate with the severity of allergic symptoms by exposure dose^(45)^. Age should also be considered since cedar pollen-specific IgE titres appear to decrease in elderly people (42). Indeed, there was a slight tendency of cedar pollen-specific IgE positive cases to be younger than negative cases (mean age, 62.3 years for negative cases and 57.6 years for positive cases). Although the participants in our data certainly tended to be older, there was a slightly stronger factor effect for age. We attempted a sub-analysis by age group under 65 and over 65, and found that a significant association between more frequent green tea intake and lower probability of cedar pollen-specific IgE positivity among <65 years of age, while no significant association was shown among ≥65 years of age. There may be some age-related immunoreactivity effects, the age of participants was around 60 years (mean, 60.3±0.9 years), it was not feasible to assess the impact of age on cedar pollen-specific IgE positivity across all age groups. Further investigation for younger population would be needed.

The strength of the present study is that it targeted a large Japanese general population and focused on the frequency of tea intake, which few studies have done in the past. We then objectively assessed cedar pollinosis, the major cause of hay fever in Japan, based on blood test data. Our findings support the beneficial effects of green tea against cedar pollinosis.

However, there are also some limitations worth noting. First, participants took part in a health survey and thus may have been relatively health conscious. Second, we obtained information on tea intake based on self-report using a questionnaire and did not have information regarding the quantity, concentration, extraction temperature, and type of tea leaves used. With regard to catechins, the amount that is present in green tea depends on the duration of steeping, temperature of the water, and plant variety. Thus, concentrations of catechins from daily tea drinking varied by individual. The green tea variety that the majority of Japanese people drink is ‘*Yabukita’*, while the variety with the highest concentration of methylated catechins hypothesised to be highly effective is ‘*Benifuki*’^(10,19,20)^. We were unable to obtain this type of compositional information for our analysis. Nevertheless, the results of the present study demonstrate a potential anti-allergic effect of frequent tea intake. Our data highlight the effectiveness of habitual once-a-day tea intake, rather than intensive intake over a certain period of time, and this daily habit may be sufficient for people to receive the beneficial effects of tea throughout the year. Based on a further analysis regarding the number of cups per day, there appeared to be no threshold for up to about 10 cups a day. As mentioned above, information regarding the quantity and concentration of tea was not available, so we cannot conclude that it is better to simply drink more tea. Third, we did not have detailed information regarding actual allergy symptoms, their severity, and the use of anti-allergy medication. In preliminary analyses, past history of allergic disease based on self-report was used as a covariate in the analytical model, but this variable did not have a considerable effect. Information regarding history of allergic disease was also incomplete because the date of onset was uncertain. Fourth, any selection bias should also be considered because about 16,000 participants were analysed in the present study out of about 60,000 cohort participants. The excluded participants from the analysis who had incomplete data on tea intake responses and allergy testing had some characteristics, such as a slightly older age; however, this did not differ substantially from the participants analysed in the present study.

The present study revealed a significant association between the frequency of tea intake and cedar pollen-specific IgE positivity. Participants who consumed green tea at least once a day had a lower odds of cedar pollen-specific IgE positivity. Further epidemiological studies to clarify the relationship between tea intake and cedar pollen allergy are warranted.

## Supporting information

Aoki et al. supplementary material Aoki et al. supplementary material

## Data Availability

The datasets generated and/or analysed during the current study are not publicly available due to a contract with Tohoku Medical Megabank Organization, but are available from the corresponding author on reasonable request.

## Supplementary material

The supplementary material for this article can be found at https://doi.org/10.1017/jns.2024.96.
