# Supplementary material for: Relationship between tea intake and cedar pollen allergy: a population-based cross-sectional study
Source: J Nutr Sci. 2025 Jan 10;14:e2. doi: 10.1017/jns.2024.96 (PMC11811857; doi:10.1017/jns.2024.96)
Supplement: Aoki et al. supplementary material [file S204867902400096Xsup001.docx]

Supplementary Table 1. Odds ratios for positive serum cedar pollen-specific IgE by frequency of green tea intake and major lifestyle variables stratified by age (age ≥65 years).

|  |  | Univariate analysis | |  | Multivariate analysis | | | |
| --- | --- | --- | --- | --- | --- | --- | --- | --- |
|  | N (%) | OR (95%CI) | *p* |  | Model 1  OR (95%CI) | *p* | Model 2  OR (95% CI) | *p* |
| Frequency of Green tea intake |  |  |  |  |  |  |  |  |
| Group 1 | 970 (13•0) | 1.00 (reference) |  |  | 1.00 (reference) |  | 1.00 (reference) |  |
| Group 2 | 1,723 (23•2) | 0•92 (0•78-1•08) | 0•35 |  | 0•92 (0•78-1•08) | 0•34 | 0•89 (0•64-1•25) | 0•51 |
| Group 3 | 4,712 (63•6) | 0•88 (0•76-1•02) | 0•09 |  | 0•92 (0•80-1•07) | 0•30 | 0•82 (0•60-1•10) | 0•19 |
| Sex, male (ref. female) | 3,157 (42•6) | - | - |  | 0•63 (0•57-0•69) | <0•01 | 0•73 (0•55-0•96) | 0•26 |
| Body mass index, ≥25 kg/m^2^ (ref.<25 kg/m^2^) | 499 (6•7) | - | - |  | - | - | 1•12 (0•90-1•41) | 0•29 |
| Lifestyle characteristics |  |  |  |  |  |  |  |  |
| Frequency of alcohol intake, daily (ref. not daily) | 617 (8•3) | - | - |  | - | - | 1•08 (0•86-1.35) | 0•49 |
| Smoking (ref. <100 cigarettes) |  |  |  |  |  |  |  |  |
| lifetime cumulative smoking of ≥100 cigarettes | 878 (11•8) | - | - |  | - | - | 1•02 (0•80-1•30) | 0•85 |
| Physical activity, walking ≥1 time/week  (ref. <1 time/week) | 568 (7•7) | - | - |  | - | - | 1•01 (0•82-1•25) | 0•89 |
| Sleep duration (ref. <6 and >8 hours/day) |  |  |  |  |  |  |  |  |
| 6-7 hours/day | 557 (7•5) | - | - |  | - | - | 0•84 (0•68-1•05) | 0•13 |
| Hypertension, medication user (ref. non-user) | 497 (6•7) | - | - |  | - | - | 1•07 (0•84-1•36) | 0•55 |
| Diabetes mellitus, medication user (ref. non-user) | 120 (1•6) | - | - |  | - | - | 0•99 (0•64-1•54) | 0•99 |
| Dyslipidemia, medication user (ref. non-user) | 195 (2•6) | - | - |  | - | - | 1•16 (0•80-1•67) | 0•42 |

Abbreviations: OR, odds ratio; 95% CI, 95% confidence interval; p, p-values; ref, reference

Group1, less than once a week; Group2, 1-6 times a week; Group3, more than once a day

The number of participants analyzed, total: 7,405, with ceder pollen allergy test negative cases: 4,806, positive cases: 2,599.

Supplementary Table 2. Odds ratios for positive serum cedar pollen-specific IgE by frequency of green tea intake and major lifestyle variables by age (age < 65 years).

|  |  | Univariate analysis | |  | Multivariate analysis | | | |
| --- | --- | --- | --- | --- | --- | --- | --- | --- |
|  | N (%) | OR (95%CI) | *p* |  | Model 1  OR (95%CI) | *p* | Model 2  OR (95% CI) | *p* |
| Frequency of Green tea intake |  |  |  |  |  |  |  |  |
| Group 1 | 2,322 (25•1) | 1.00 (reference) |  |  | 1.00 (reference) |  | 1.00 (reference) |  |
| Group 2 | 3,029 (32•8) | 0•93 (0•84-1•04) | 0•25 |  | 0•93 (0•84-1•04) | 0•25 | 0•95 (0•80-1•13) | 0•60 |
| Group 3 | 3,867 (41 •9) | 0•75 (0•68-0•84) | <0•01 |  | 0•76 (0•68-0•84) | <0•01 | 0•81 (0•68-0•97) | 0•02 |
| Sex, male (ref. female) | 2,843 (30•8) | - | - |  | 0•91 (0•83-0•99) | 0•04 | 1•00 (0•85-1•18) | 0•96 |
| Body mass index, ≥25 kg/m^2^ (ref.<25 kg/m^2^) | 932 (10•1) | - | - |  | - | - | 1•00(0•85-1•17) | 0•98 |
| Lifestyle characteristics |  |  |  |  |  |  |  |  |
| Frequency of alcohol intake, daily (ref. not daily) | 1,069 (11•5) | - | - |  | - | - | 0•93(0•79-1•09) | 0•37 |
| Smoking (ref. <100 cigarettes) |  |  |  |  |  |  |  |  |
| lifetime cumulative smoking of ≥100 cigarettes | 1,810 (19•6) | - | - |  | - | - | 0•94 (0•80-1•10) | 0•45 |
| Physical activity, walking ≥1 time/week  (ref. <1 time/week) | 607 (6•9) | - | - |  | - | - | 1•05 (0•87-1•25) | 0•58 |
| Sleep duration (ref. <6 and >8 hours/day) | 1,384 (15•0) |  |  |  |  |  |  |  |
| 6-7 hours/day |  | - | - |  | - | - | 1•03 (0•90-1•19) | 0•60 |
| Hypertension, medication user (ref. non-user) | 468 (5•1) | - | - |  | - | - | 1•39 (1•12-1•73) | 0•02 |
| Diabetes mellitus, medication user (ref. non-user) | 85 (0•9) | - | - |  | - | - | 0•76 (0•47-1•22) | 0•25 |
| Dyslipidemia, medication user (ref. non-user) | 171 (1•9) | - | - |  | - | - | 1•08 (0•77-1•52) | 0•63 |

Abbreviations: OR, odds ratio; 95% CI, 95% confidence interval; p, p-values; ref, reference

Group1, less than once a week; Group2, 1-6 times a week; Group3, more than once a day

The number of participants analyzed, total: 9,218, with ceder pollen allergy test negative cases: 4,502, positive cases: 4,716.
